# Practice of Integrated Volitional Control Electrical Stimulation in a Patient With Post-stroke Upper Limb Paresis and Difficulty in Thumb Opposition: A Case Report

**DOI:** 10.7759/cureus.101830

**Published:** 2026-01-19

**Authors:** Takashi Nakamori

**Affiliations:** 1 Department of Occupational Therapy, Faculty of Health Sciences, Kansai University of Health Sciences, Osaka, JPN

**Keywords:** electrical stimulation, fugl–meyer assessment, ives, stroke, thumb opposition, upper-limb paresis

## Abstract

Upper limb paresis after stroke often limits functional grasp and fine manipulation, particularly owing to impaired palmar abduction and opposition of the thumb. Integrated volitional control electrical stimulation (IVES) synchronizes voluntary muscle activity with electrical stimulation and may promote motor learning and cortical reorganization. However, most applications have focused on facilitating finger extension, and approaches specifically targeting active thumb movement remain limited. This case report describes an IVES-based intervention designed to promote thumb palmar abduction during voluntary finger extension. A 40-year-old right-handed man presenting with left hemiplegia underwent outpatient occupational therapy using IVES in power-assist mode. Electrodes were placed over the extensor digitorum communis and thenar muscles to induce synchronized finger extension and thumb palmar abduction. Task-oriented training simulating motorcycle maintenance tasks was performed for 60 min, three times per week, for 10 weeks. After the intervention, upper limb motor function and activity level outcomes improved. The Fugl Meyer Assessment Upper Extremity score increased from 37 to 47 points, the Simple Test for Evaluating Hand Function score increased from 11 to 30 points, and the grip strength of the affected hand increased from 0.5 to 6.0 kg. Additionally, Motor Activity Log scores improved, and the patient reported clinically meaningful gains in bimanual motorcycle maintenance performance on the Canadian Occupational Performance Measure. This case suggests that synchronizing thumb palmar abduction with voluntary finger extension using IVES may facilitate active motor learning and functional recovery of grasp patterns requiring thumb opposition, offering an active alternative to passive assistance approaches.

## Introduction

Stroke can cause paresis of the upper limbs, leading to limitations in activities of daily living (ADLs) and a decline in quality of life [1]. Upper limb motor impairment occurs in approximately 70% of patients after stroke, and such impairments often persist, resulting in limitations in functional recovery [2]. Among various rehabilitation techniques, neuromuscular electrical stimulation is widely used for promoting voluntary movement and functional recovery in patients with stroke. Specifically, integrated volitional control electrical stimulation (IVES) delivers electrical stimulation in response to the electromyographic activity generated by voluntary muscle contraction, thereby synchronizing movement intention with motor output [3]. In this system, electrical stimulation is provided only when the patient actively attempts to move, allowing the integration of voluntary effort and sensory feedback during training. This technique promotes motor learning through repetitive sensorimotor coupling and induces cortical reorganization [4].

In clinical practice, surface electrodes are typically placed on the extensor digitorum communis and other finger extensors to facilitate their extension. These interventions improve voluntary finger extension movements and upper limb function in patients with hemiplegia [5,6]. However, clinical observations of post-stroke upper limb paresis indicate that impaired finger extension and thumb motor paresis often restrict palmar abduction and opposition movements. These limitations can hinder functional grasping and fine manipulation. The thumb accounts for approximately 40% of the total hand function; acquiring opposition is essential for dexterous movements and independence in daily activities [7].

Splints that maintain or assist thumb opposition are commonly used to address restrictions in thumb movement [8,9]. Although these splints help secure proper thumb positioning, they only provide passive assistance and cannot be considered as a direct means of promoting active motor learning or neural reorganization. Therefore, there is a clinical need for an active task-oriented rehabilitation training approach that promotes voluntary thumb movement and functional grasping.

In this case, changes in motor impairment, hand dexterity, spasticity, real-world arm use, and patient-perceived performance were evaluated using several standardized outcome measures. Motor impairment of the upper limb was assessed using the Fugl-Meyer Assessment-Upper Extremity (FMA-UE) [10], and hand dexterity was assessed using the Simple Test for Evaluating Hand Function (STEF) [11]. Spasticity of the affected upper limb was graded using the Modified Ashworth Scale (MAS) [12]. Real-world use of the paretic arm was evaluated with the motor activity log (MAL) [13], and patient-perceived performance and satisfaction regarding the target activity of bimanual motorcycle maintenance were assessed using the Canadian Occupational Performance Measure (COPM) [14].

This case report aimed to describe an intervention using IVES, with a specific electrode placement approach, to facilitate active thumb palmar abduction synchronized with voluntary finger extension in a patient with post-stroke upper limb paresis.

## Case presentation

Patient

A 40-year-old right-handed man, who had been previously healthy, presented with weakness of the left upper and lower extremities. The patient was diagnosed with cerebral infarction affecting the right putamen and corona radiata based on MRI at an acute care hospital. The day of stroke onset was defined as day X, and subsequent days were expressed as X+n (e.g., X+101 indicates 101 days after stroke onset).

Stroke severity at onset was not assessed using the National Institutes of Health Stroke Scale (NIHSS), and NIHSS data were not available in the referral document. The patient received conservative treatment and rehabilitation and was discharged 80 days post-stroke. At home, the patient independently performed ADLs, achieving a Barthel Index score of 100 [15]. Regarding medication use, the patient continued receiving nifedipine, olmesartan, and aspirin for blood pressure control and secondary stroke prevention; no pharmacological treatments directly affecting upper limb motor function or spasticity were administered. Outpatient occupational therapy at our hospital commenced on day X+101.

Baseline assessment

The pre-intervention assessment was conducted on day X+101. The total FMA-UE score was 37/66 points, indicating moderate paresis of the affected upper limb. Based on the baseline FMA-UE score, the severity of upper limb paresis was classified as moderate, according to previously reported criteria [16]. Grip strength was measured as 37 kg on the right and 0.5 kg on the left. The MAS showed a grade of 1+ for the flexors of the fingers, wrist, and elbow, indicating mild spasticity.

Hand dexterity was assessed using the STEF, a standardized assessment of upper limb function developed in Japan. The participants moved various objects to designated positions as quickly as possible, and the time taken was converted into a score of 0-100 points [11]. In this case, the right hand had a score of 100 points, and the left hand had 11 points. The average score for healthy individuals in their 40s is approximately 100 points. Tasks requiring dexterity, such as handling clothes, small balls, and pins, were difficult to complete, and the patient could not complete them within the time limit. Opposing the thumb was particularly challenging, as the patient primarily used a lateral grasp pattern for these tasks. The MAL showed low scores (1.4 for both amount of use (AOU) and quality of movement (QOM)), indicating limited use of the affected hand in daily life. Sensory and cognitive functions were normal. Using the COPM, the patient expressed the desire to “maintain my motorcycle using both hands.” The subjective assessments of the activity were important (10/10), performance (2/10), and satisfaction (2/10). The patient reported difficulty in grasping objects with the left hand and said that they used it only to lightly stabilize objects. Most maintenance tasks were performed primarily using the right hand.

Intervention

Occupational therapy interventions were conducted on an outpatient basis using IVES+ (Model GD611, OG Wellness Co., Ltd., Japan) in the power-assist mode for electrical stimulation [5,17]. The IVES+ consists of a two-pole electrode (46×52 mm) that detects electromyography (EMG) and delivers electrical output, and a one-pole electrode (46×46 mm) that delivers electrical output. EMG detection was performed solely with the bipolar electrode, and electrical stimulation was performed between the bipolar and unipolar electrodes.

**Figure 1:**
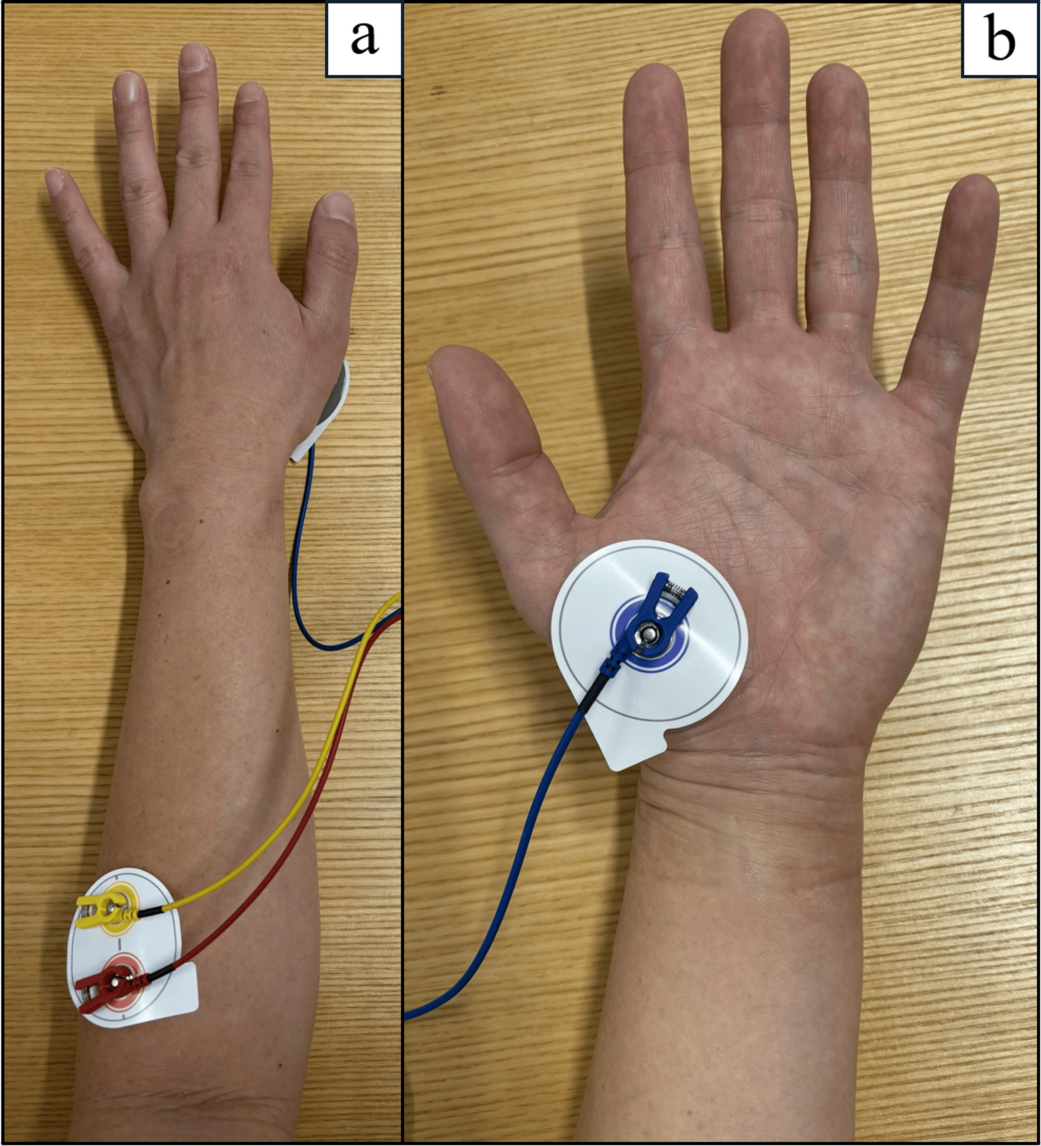
Electrode placement for IVES+ (Model GD611, OG Wellness Co., Ltd., Japan) (a) Two-pole electrode positioned over the extensor digitorum communis on the dorsal aspect of the left forearm. (b) Single-pole electrode positioned over the thenar eminence on the palmar aspect of the left hand.

The EMG detection electrode was placed over the extensor digitorum communis muscle on the affected forearm, and the stimulation output electrode was placed over the thenar muscles. The system was configured such that, upon detecting EMG activity from the finger extensor muscles, electrical stimulation was delivered simultaneously to the extensor digitorum communis and thenar muscles (Figure 1). The stimulation parameters were set to a frequency of 20 Hz and a pulse width of 50 μs. Furthermore, the minimum intensity was set below the motor threshold, where stimulation was perceptible but did not produce visible muscle contraction. The maximum intensity was set at the motor threshold, where muscle contraction was observable without discomfort and resulted in finger extension and thumb abduction. Electrical stimulation was delivered in proportion to voluntary muscle activity in the left hand, ranging from minimum to maximum intensity.

Each 60-min session was conducted three times per week for 10 weeks. A qualified occupational therapist performed all the sessions. Each session involved task-oriented training that simulated actual motorcycle maintenance tasks [18]. These tasks included rotating nuts with pliers or a screwdriver, manipulating small objects, and performing pinching actions with the thumb and either the index or middle finger. Additionally, activities that closely resembled actual maintenance tasks, such as grasping and transferring small parts, were incorporated. The training emphasized coordinated voluntary finger extension and thumb palmar abduction to encourage functional grasping.

The patient concurrently received standard physical therapy three times per week, focusing on lower-limb muscle strengthening, gait training, and balance exercises. These sessions did not include direct intervention for the upper limbs. The occupational therapy intervention was planned and conducted in consultation with the treating physical therapist to ensure consistency with the overall rehabilitation program.

Results

**Figure 2:**
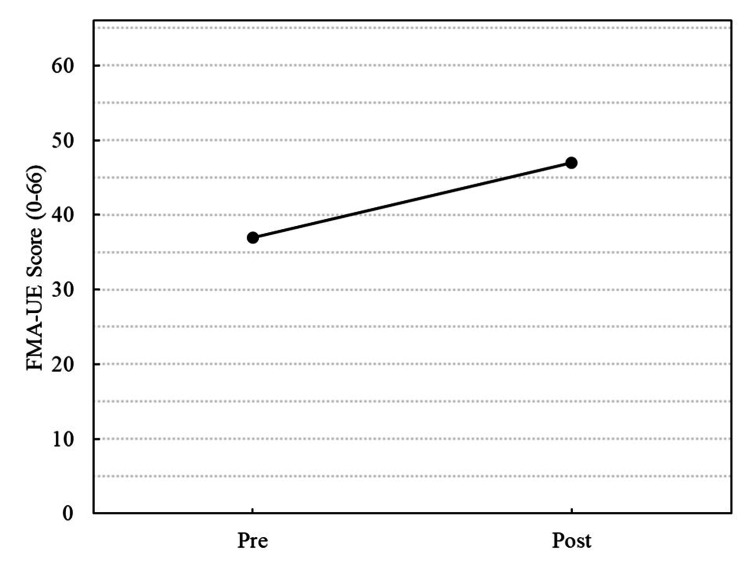
Changes in the FMA-UE total score from baseline (Day X+101) to post-intervention (Day X+173)

Reassessment was conducted on Day X+173 (Table 1, Figure 2). All evaluation items based on left upper limb motor function and performance showed significant improvements compared with the baseline. Specifically, the FMA-UE score increased from 37 to 47, the STEF score increased from 11 to 30, and the grip strength increased from 0.5 to 6.0 kg. The AOU and QOM for the MAL both improved from 1.4 to 2.8. Additionally, the COPM performance and satisfaction scores for the goal of performing motorcycle maintenance with both hands increased from 2 to 6. The MAS score remained unchanged at 1+ for all items, and no worsening of spasticity was observed during the intervention period.

**Table 1:** Changes in the upper limb motor function and activity-level outcomes from baseline (Day X+101) to the post-intervention period (Day X+173) For FMA-UE, each subscore (A–D) and the total score are expressed as “patient score/maximum possible score” (e.g., 27/36). FMA-UE, Fugl–Meyer Assessment-Upper Extremity; MAS, Modified Ashworth Scale; STEF, Simple Test for Evaluating Hand Function; MAL, Motor Activity Log; AOU, Amount of Use; QOM, Quality of Movement; COPM, Canadian Occupational Performance Measure; R, right; L, left

Variables		Pre（X+101）	Post（X+173）
FMA-UE (point)	A: Shoulder/Elbow	27/36	29/36
	B: Wrist	4/10	5/10
	C: Hand	6/14	10/14
	D: Coordination/ Speed	0/6	3/6
	Total	37/66	47/66
Grip strength (R/L) (kg)		37/0.5	37/6
MAS (point)		1+	1+
STEF (R/L) (point)		100/11	100/30
MAL (point)	AOU	1.4	2.8
	QOM	1.4	2.8
COPM (point)	Performance	2/10	6/10
	Satisfaction	2/10	6/10

The patient gained the ability to spontaneously perform palmar abduction and opposition of the thumb without electrical stimulation. This enabled fingertip pinching, which requires thumb opposition. During work activities, the patient demonstrated more coordinated grasping patterns and achieved a functional level of performance in two-handed motorcycle maintenance tasks. The patient stated, “I can do more things with both hands, like electrical work, and it's good that my efficiency has improved.” No adverse events, such as pain, skin irritation, or muscle fatigue, were observed during the program. The intervention was completed safely without any complications.

## Discussion

In this case, synchronizing thumb palmar abduction with voluntary finger extension using the IVES technique improved thumb opposition function and overall functional performance. Before the intervention, the patient could not perform thumb palmar abduction or opposition movements and was limited to lateral pinching, which made object manipulation difficult. After the intervention, the FMA-UE score increased by 10 points, specifically by 4 points in the hand subscore. Additionally, the STEF increased by 19 points, enabling grasping patterns that require thumb opposition, such as fingertip pinching. These results suggest that coupling electrical stimulation with voluntary movement may promote sensorimotor integration and facilitate motor learning during task-oriented training.

IVES promotes cortical reorganization and motor learning by synchronizing motor intention with sensory input from electrical stimulation [3,19,20]. Previous studies have targeted the extensor digitorum communis muscle and reported improvements in voluntary hand opening and extension movements by facilitating finger extension [5,6,17]. Conversely, applications primarily aimed at promoting palmar abduction or opposition of the thumb remain relatively scarce [21]. This case is novel because it elicited difficult-to-induce thumb movement patterns by devising a novel electrode placement, which led to improved opposition movement.

Acquiring thumb opposition is essential for dexterous movements and practical hand use in daily life [22]. Traditionally, methods have involved guiding the thumb into opposition using devices such as thumb opposition splints, followed by training in that position [8]. However, although these methods are useful for thumb positioning, they provide only passive assistance and cannot be considered as interventions that directly promote active motor learning or sensorimotor integration. In this study, the electrodes were placed on the extensor digitorum communis and thenar muscles. These were configured to induce active palmar thumb abduction during finger extension. This synchronized movement likely contributed to the functional recovery by enabling repeated object manipulation in a physiologically appropriate hand posture.

Furthermore, through repeated IVES-assisted training combined with task-oriented practice, the patient regained multiple grasping patterns that required thumb opposition. These patterns included grasping spherical and cylindrical objects and lateral pinching. These changes likely reflect increased muscle strength and reconstruction of coordinated movements between the thumb and other fingers [6,17]. Repetitive sensorimotor input via electrical stimulation synchronized with voluntary movement may have promoted cortical-level neuroplasticity, supporting recovery from palmar abduction and opposition of the thumb [19,20].

According to the manual and previous studies, a change of approximately two points or more on the COPM is considered clinically meaningful [14,23]. Therefore, the four-point improvement in performance and satisfaction observed in this case was considered a clinically significant change. This aligns with the patient's statements and can be interpreted as a meaningful subjective improvement, as the patient gained the ability to grasp with the left hand and perform bimanual tasks.

## Conclusions

The results of this case suggest that the electrode placement approach used in this intervention may support active motor learning, distinct from passive assistance strategies. This method has high potential for clinical application, particularly for patients with moderate upper limb paresis, as it allows repetitive practice of functionally relevant tasks while maintaining appropriate movement patterns. Furthermore, it may promote reactivation of the thumb abductor muscle group and contribute to improved coordination of the entire upper limb.

However, this report is based on a single case, which limits the generalizability of the findings. In addition, no long-term follow-up assessment was conducted after the intervention period. Future studies should verify reproducibility across multiple cases using similar electrode placements and settings. In addition, long-term follow-up evaluations are necessary to determine whether improvements in thumb motor functions are sustained over time and to assess generalization to ADLs and social participation.

## References

[1] O'Flaherty D, Ali K (2024). Recommendations for upper limb motor recovery: an overview of the UK and European rehabilitation after stroke guidelines (2023). Healthcare (Basel).

[2] Nakayama H, Jørgensen HS, Raaschou HO, Olsen TS (1994). Recovery of upper extremity function in stroke patients: the Copenhagen stroke study. Arch Phys Med Rehabil.

[3] Hara Y (2008). Neurorehabilitation with new functional electrical stimulation for hemiparetic upper extremity in stroke patients. J Nippon Med Sch.

[4] Shin HK, Cho SH, Jeon HS (2008). Cortical effect and functional recovery by the electromyography-triggered neuromuscular stimulation in chronic stroke patients. Neurosci Lett.

[5] Yamaguchi T, Tanabe S, Muraoka Y (2011). Effects of integrated volitional control electrical stimulation (IVES) on upper extremity function in chronic stroke. Keio J Med.

[6] Fujiwara T, Kasashima Y, Honaga K (2009). Motor improvement and corticospinal modulation induced by hybrid assistive neuromuscular dynamic stimulation (HANDS) therapy in patients with chronic stroke. Neurorehabil Neural Repair.

[7] Emerson ET, Krizek TJ, Greenwald DP (1996). Anatomy, physiology, and functional restoration of the thumb. Ann Plast Surg.

[8] Amano S, Takebayashi T, Hanada K, Umeji A, Marumoto K, Furukawa K, Domen K (2015). Constraint-induced movement therapy after injection of botulinum toxin type A for a patient with chronic stroke: one-year follow-up case report. Phys Ther.

[9] Ali IB, Elshazly FA, Ali MS (2022). The effect of a thumb web spacer splint on hand function in children with hemiplegic cerebral palsy. J Taibah Univ Med Sci.

[10] Fugl-Meyer AR, Jääskö L, Leyman I, Olsson S, Steglind S (1975). The post-stroke hemiplegic patient. 1. A method for evaluation of physical performance. Scand J Rehabil Med.

[11] Kaneko T, Muraki T (1990). Development and standardization of the hand function test. BAMS (Kobe).

[12] Bohannon RW, Smith MB (1987). Interrater reliability of a modified Ashworth scale of muscle spasticity. Phys Ther.

[13] Uswatte G, Taub E, Morris D, Vignolo M, McCulloch K (2005). Reliability and validity of the upper-extremity motor activity log-14 for measuring real-world arm use. Stroke.

[14] Law MC, Carswell A, Baptiste S, McColl MA, Polatajko H, Pollock N (2019). Canadian Occupational Performance Measure (COPM), 5th Edition. Canadian Occupational Performance Measure: COPM.

[15] Mahoney FI, Barthel D (1965). Functional evaluation: the Barthel index. Md State Med J.

[16] Woodbury ML, Velozo CA, Richards LG, Duncan PW (2013). Rasch analysis staging methodology to classify upper extremity movement impairment after stroke. Arch Phys Med Rehabil.

[17] Hara Y, Ogawa S, Tsujiuchi K, Muraoka Y (2008). A home-based rehabilitation program for the hemiplegic upper extremity by power-assisted functional electrical stimulation. Disabil Rehabil.

[18] Winstein CJ, Wolf SL, Dromerick AW (2016). Effect of a task-oriented rehabilitation program on upper extremity recovery following motor stroke: the ICARE randomized clinical trial. JAMA.

[19] Hara Y, Obayashi S, Tsujiuchi K, Muraoka Y (2013). The effects of electromyography-controlled functional electrical stimulation on upper extremity function and cortical perfusion in stroke patients. Clin Neurophysiol.

[20] Hara Y (2013). Rehabilitation with functional electrical stimulation in stroke patients. Int J Phys Med Rehabil.

[21] Arantes NF, Vaz DV, Mancini MC, Pereira MS, Pinto FP, Pinto TP (2007). Effects of functional electrical stimulation applied to the wrist and finger muscles of hemiparetic subjects: a systematic review of the literature. Braz J Phys Ther.

[22] Parry R, Macias Soria S, Pradat-Diehl P, Marchand-Pauvert V, Jarrassé N, Roby-Brami A (2019). Effects of hand configuration on the grasping, holding, and placement of an instrumented object in patients with hemiparesis. Front Neurol.

[23] Ohno K, Tomori K, Sawada T, Kobayashi R (2021). Examining minimal important change of the Canadian occupational performance measure for subacute rehabilitation hospital inpatients. J Patient Rep Outcomes.

